# Modulation of electrical potential and conductivity in an atomic-layer semiconductor heterojunction

**DOI:** 10.1038/srep31223

**Published:** 2016-08-12

**Authors:** Yu Kobayashi, Shoji Yoshida, Ryuji Sakurada, Kengo Takashima, Takahiro Yamamoto, Tetsuki Saito, Satoru Konabe, Takashi Taniguchi, Kenji Watanabe, Yutaka Maniwa, Osamu Takeuchi, Hidemi Shigekawa, Yasumitsu Miyata

**Affiliations:** 1 Department of Physics, Tokyo Metropolitan University, Hachioji, Tokyo 192-0397, Japan; 2 Faculty of Pure and Applied Sciences, University of Tsukuba, Tsukuba, Ibaraki 305-8573, Japan; 3 Department of Electrical Engineering, Graduate School of Engineering, Tokyo University of Science, Katsushika, Tokyo 125-8585, Japan; 4 Department of Liberal Arts, Faculty of Engineering, Tokyo University of Science, Katsushika, Tokyo 125-8585, Japan; 5 Research Institute for Science and Technology, Tokyo University of Science, Katsushika, Tokyo 125-8585, Japan; 6 National Institute for Materials Science, Tsukuba, Ibaraki 305-0044, Japan; 7 JST-PRESTO, Kawaguchi, Saitama 332-0012, Japan

## Abstract

Semiconductor heterojunction interfaces have been an important topic, both in modern solid state physics and in electronics and optoelectronics applications. Recently, the heterojunctions of atomically-thin transition metal dichalcogenides (TMDCs) are expected to realize one-dimensional (1D) electronic systems at their heterointerfaces due to their tunable electronic properties. Herein, we report unique conductivity enhancement and electrical potential modulation of heterojunction interfaces based on TMDC bilayers consisted of MoS_2_ and WS_2_. Scanning tunneling microscopy/spectroscopy analyses showed the formation of 1D confining potential (potential barrier) in the valence (conduction) band, as well as bandgap narrowing around the heterointerface. The modulation of electronic properties were also probed as the increase of current in conducting atomic force microscopy. Notably, the observed band bending can be explained by the presence of 1D fixed charges around the heterointerface. The present findings indicate that the atomic layer heterojunctions provide a novel approach to realizing tunable 1D electrical potential for embedded quantum wires and ultrashort barriers of electrical transport.

Semiconductor heterojunctions are vital components in modern solid-state physics and have applications in electronics and optoelectronics. As an example, conventional three-dimensional heterojunctions of crystalline semiconductors, such as GaAs/AlGaAs, can generate a high-mobility two-dimensional electron gas (2DEG) at their two-dimensional interface^1^,^2^,^3^. The realization of clean 2DEG in this manner has led to the discovery of exotic physical phenomena, including fractional quantum Hall effects^4^,^5^, and to practical applications in the channels within high-performance field-effect transistors^2^. A high mobility 2DEG and interface superconductivity have been also found at the heterointerfaces of oxides^6^,^7^. Recently, similar heterojunctions consisting of so-called atomic layers have been fabricated in two-dimensional crystals, and have attracted much attention based on their unique low-dimensional structures and physical properties^8^,^9^,^10^,^11^,^12^,^13^,^14^,^15^,^16^,^17^,^18^. The in-plane, lateral heterojunctions employed in initial studies were composed of metallic graphene and insulating hexagonal boron nitride (BN) monolayers^8^,^9^,^10^,^11^,^12^. Atomic layer-based semiconductor heterojunctions such as these are expected to represent ideal systems for the realization of novel confined electronic systems at one-dimensional (1D) interfaces, and should lead to novel physics and electronic/optoelectronics discoveries.

Several groups have reported the synthesis of semiconductor heterojunctions based on transition metal dichalcogenides (TMDCs) by vapor phase growth techniques^13^,^14^,^15^,^16^,^17^. TMDCs are ideal materials for semiconductor heterojunctions because of their unique compositions, polytypes and layer number-dependent electronic properties^19^,^20^. In early studies, these heterojunctions were composed primarily of Mo- and W-based semiconductor TMDCs, such as MoS_2_, MoSe_2_, WS_2_ and WSe_2_ monolayers^13^,^14^,^15^. In a previous paper, we demonstrated the use of similar lateral heterostructures employing monolayers of Mo_1−x_W_x_S_2_ alloys, which have composition-dependent tunable bandgaps^16^,^17^. These earlier studies generated band diagrams by scanning tunneling microscopy/spectroscopy (STM/STS), and showed that such monolayer heterointerfaces result in staggered type-II heterojunctions in which there is no potential barrier for carrier confinement^17^. Despite these significant earlier works, there have been no reports to date of the conducting 1D interface and 1D confining potential/potential barriers associated with these TMDC heterojunctions, likely because these prior works have focused solely on monolayer-based heterojunctions.

In the present study, we observed conductivity enhancement and unique band bending at the interface of TMDC bilayer-based heterojunctions. These heterojunctions were composed of WS_2_ bilayers and vertically stacked WS_2_/MoS_2_ heterostructures, grown by two-step chemical vapor deposition (CVD). The conductivity enhancement was assessed by conducting atomic force microscopy (c-AFM). Furthermore, STM/STS measurements demonstrated an upshift in both the valence and conduction band edges, as well as band-gap narrowing around the heterointerface. These results indicate that the highly tunable electronic properties of TMDCs provide an ideal system allowing the realization of tunable 1D electronic systems at the heterointerfaces between atomic layers.

## Results

**Figure 1:**
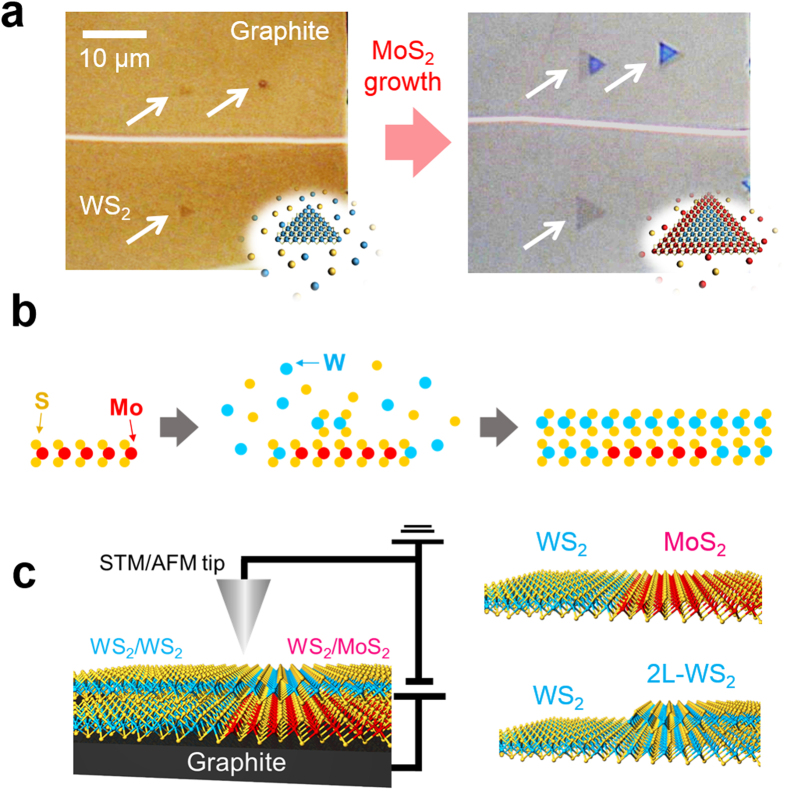
Growth and structures of three types of heterojunctions based on WS_2_ and MoS_2_. (**a**) Optical images and illustrations for a sample before and after MoS_2_ growth around WS_2_ grains on graphite. (Note: in the structural model, cyan, red and yellow correspond to W, Mo and S atoms, respectively.) (**b**) Illustration of a growth model for a bilayer heterojunction based on bilayer WS_2_ - stacked WS_2_/MoS_2_. (**c**) Structures and measurement setup for the three heterojunctions studied in the present work.

High-temperature CVD was used to prepare high-quality TMDC samples on graphite and boron nitride flakes, as reported in our previous paper^21^. This process forms mono- and multilayer grains of MoS_2_ and WS_2_ with sizes around 10 μm. As shown in Fig. 1a, WS_2_ (or MoS_2_) grains are initially synthesized on the graphite, after which the second growth process of another TMDCs is conducted with firstly-grown grains after changing the reaction chamber to avoid alloy formation, in agreement with a previous report by Li *et al*.^18^. In addition to the formation of a conventional monolayer heterojunction, we also found that a second TMDC layer can simultaneously grow both from the grain edges and on the grain surfaces, leading to the formation of bilayer TMDC-based heterojunctions, as shown in Fig. 1b. We note that only the initial monolayers formed on the graphite or BN tend to survive, and that these same monolayers grown on SiO_2_ or sapphire substrates easily degrade during the second growth process at 1100 °C. These findings indicate that the stability of WS_2_ and MoS_2_ is sensitive to the growth substrate, and is a critical factor in the second growth. In the present work, we investigated three types of heterojunctions, including monolayer WS_2_ and MoS_2_ (1L WS_2_-MoS_2_), bilayer WS_2_ - stacked WS_2_/MoS_2_ (2L WS_2_-WS_2_/MoS_2_) and monolayer and bilayer WS_2_ (1L-2L WS_2_), using c-AFM and STM/STS, as shown in Fig. 1c.

**Figure 2:**
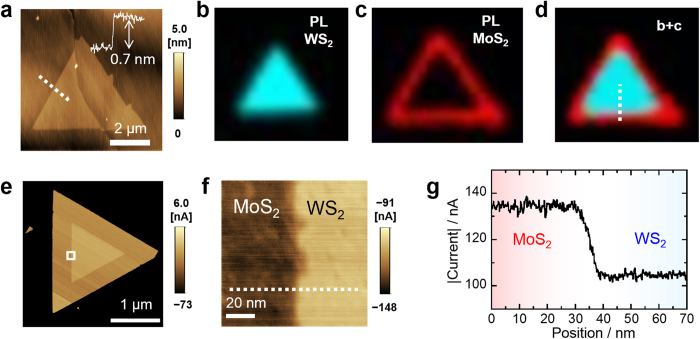
Characterization of a heterojunction based on monolayer WS_2_ and MoS_2_. (**a**) AFM image and height profile of a triangle-shaped grain with a monolayer WS_2_/MoS_2_ lateral heterostructure grown on graphite. The height profile was obtained along the dotted line. (**b–d**) PL intensity maps from 1.92 to 1.99 eV for WS_2_ and 1.80 to 1.88 eV for MoS_2_, and a combined PL intensity map of (**b**,**c**). Cyan and red correspond to the intensities of the WS_2_ and MoS_2_ PL peaks, respectively. (**e**,**f**) Current images of the grain and the expanded area indicated by the white box in (**e**). (**g**) Current profile along the dotted line in (**f**).

A model of the heterostructures composed of a triangular WS_2_ monolayer with lateral MoS_2_ is shown in Fig. 1a. In this sample, the inner and outer parts correspond to WS_2_ and MoS_2_ monolayers, respectively, as confirmed by AFM images and a height profile (Fig. 2a), as well as the photoluminescence (PL) intensity maps (Fig. 2b–d) and Raman data (Supplementary Fig. S1). The PL maps show that the WS_2_ is primarily located in the inner part of the crystal, whereas the MoS_2_ is formed around the inner WS_2_. Almost the same structure is indicated by the Raman intensity maps of WS_2_ and MoS_2_. In the PL and Raman spectra (Supplementary Fig. S2), both the excitonic PL peaks and vibrational modes of WS_2_ and MoS_2_ are observed around the interface, with no peak shift or broadening caused by alloy formation. Furthermore, the PL peaks exhibit symmetric Lorentzian profiles and narrow linewidths, with full width at half maximum (FWHM) values of 20 and 47 meV for the WS_2_ and MoS_2_ (Supplementary Fig. S3), respectively, indicating high-quality samples^21^.

In addition to the optical measurements, the electrical properties of the monolayer WS_2_/MoS_2_ heterojunction were investigated, using c-AFM in air. To obtain a current map, the conducting tip was scanned along a TMDC specimen grown on a graphite substrate, which served as an electrode (Fig. 1c). As shown in Fig. 2e,f, this technique allows visualization of the difference in the vertical electrical conductivities between the inner WS_2_ and the outer MoS_2_. The enlarged current image indicates a straight, steep heterojunction interface (Fig. 2f). Interestingly, the current profile reveals that the current value abruptly changes from 135 nA in the MoS_2_ to 115 nA in the WS_2_ within a region of 8 nm around the interface (Fig. 2g). In contrast, there are no differences in the topographic image and height profile in the same area (Supplementary Fig. S4). The continuous change in current suggests that the bandgap gradually changes in the vicinity of the heterointerface, and that there is no large band offset as reported in our previous STM/STS study of monolayer-based lateral heterostructures^17^.

A similar current profile was obtained for the 1L-2L WS_2_ heterojunction. As shown in Supplementary Fig. S5a,b, additional triangular WS_2_ monolayers were occasionally grown on the surfaces of large triangular WS_2_ monolayers during the CVD process. The current image and profile of the grain also demonstrate a difference in conductivity between the 1L and 2L WS_2_ (Supplementary Fig. S5c,d). In the profile, the current changes continuously from 140 nA in the 2L region to 80 nA in the 1L region. This strongly suggests that the spatial changes in the bandgap of the 1L-2L WS_2_ heterojunction are similar to those that occur in monolayer heterojunctions and that the edge state of the top layer has only a limited effect on the present current measurements in air.

**Figure 3:**
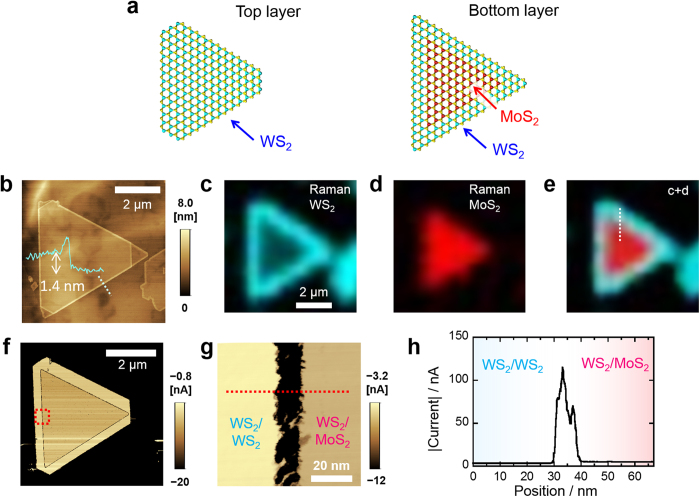
Characterization of a heterojunction based on bilayer TMDCs. (**a**) Illustration of the top and bottom layers of the present bilayer TMDC grains. (**b**) Topographic AFM image and height profile of the bilayer grain grown on graphite. (**c**,**d**) Raman intensity maps of WS_2_ (2LA and E’ modes) and MoS_2_ (E’ mode). (**e**) Combined Raman intensity map of (**c**,**d**). (**f,g**) Current images of the grain and the expanded area indicated by the dotted box in (**f**). Note that the color is adjusted to be black for the current values less than −20 for (**f**) and −12 for (**g**) to enhance the contrast between the inner and outer regions. (**h**) Current profile along the dotted line in (**g**).

In contrast, drastic current enhancement at the heterointerface is observed in the case of the bilayer-based heterojunctions. In the present bilayer samples, WS_2_ was grown from the edge and on the surfaces of monolayer MoS_2_ grains, as illustrated in Figs 1b and 3a. Both the AFM image and the height profile show triangular crystals approximately 5 μm in size and with heights of 1.4 nm, corresponding to the thickness of the bilayer TMDC (Fig. 3b). The Raman intensity maps (Fig. 3c–e) and spectra (Supplementary Fig. S6) of the same crystal exhibit a MoS_2_ peak only in the inner part of the crystal. Conversely, WS_2_ peaks were generated for both the inner and outer parts and have a particularly high intensity in the outer part. The PL map and spectra (Supplementary Fig. S7) indicate only monolayers around the corners, which generate sharp PL peaks, while no signal is observed for the bilayer part grown on graphite. This can be understood as a result of the indirect bandgap nature of both the 2L WS_2_ (refs 22,23) and MoS_2_/WS_2_ vertical heterostructures (refs 24, 25, 26), and the presence of fast non-radiative decay processes on the graphene surface^21^. These results demonstrate the formation of lateral and vertical heterostructures of MoS_2_ and WS_2_, as presented in Fig. 3a. Importantly, these heterostructures can be regarded as the lateral heterojunction of the outer 2L WS_2_ and inner WS_2_/MoS_2_ vertically stacked heterostructures.

The bandgaps of these two different bilayer TMDCs were probed by acquiring PL spectra of samples formed on bulk hexagonal boron nitride (BN) flakes in place of graphite as the substrate (Supplementary Fig. S8). In the case of the 2L WS_2_ grown on BN flakes, an indirect PL peak can be observed at 1.8 eV, which has been also observed for samples on SiO_2_^22^,^23^. The WS_2_/MoS_2_ vertical heterostructures produced three additional peaks over the range of 1.5 to 1.7 eV in addition to the PL peaks observed for monolayer MoS_2_ (1.87 eV) and WS_2_ (2.03 eV). We tentatively assign these new peaks to interlayer excitons associated with electron and hole pairs having different momenta, such as K-K and K-Γ points^24^,^25^,^26^. We note that only a single broad peak has been reported for MoS_2_/WS_2_ vertical heterostructures grown on SiO_2_^13^. These observations indicate that the inner and outer bilayer parts have different bandgaps, and so the sample corresponds to a semiconductor heterojunction based on bilayer TMDCs.

Unlike the current images of the 1L MoS_2_/WS_2_ and 1L-2L WS_2_, the bilayer grain exhibits black lines with a triangular shape along the interface (Fig. 3f,g), indicating that a significant current can flow across the bilayer from the tip to the graphite substrate. For the in-plane parts, the inner triangle regions exhibit somewhat higher current values than the outer parts of the sample, which is consistent with the different electronic structures of these portions, as observed in the PL spectra. Interestingly, the current is enhanced by several tens of times over a 10 nm region around the heterointerface as compared to the in-plane bilayers (Fig. 3h). We note that the same results have been obtained with other bilayer grains (Supplementary Fig. S9). These conducting 1D interfaces are a unique feature in the present bilayer heterojunctions, and are observed for the first time ever in the present study.

**Figure 4:**
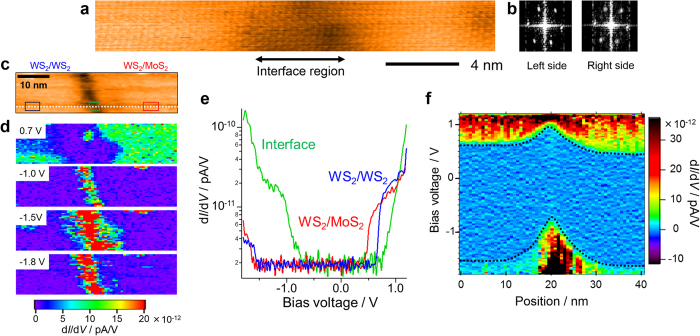
STM and STS study of a heterojunction based on bilayer TMDCs. (**a**) High-resolution STM image around the bilayer-based heterointerface (*V*_s_ = +1.4 V, *I*_t_ = 10 pA). (**b**) FFT images of left and right sides of (**a**). (**c**) Wide-area STM image around the bilayer heterointerface (*V*_s_ = +1.2 V, *I*_t_ = 10 pA). (**d**) d*I*/d*V* maps at different sample bias voltages of *V*_s_ = 0.7, −1.0, −1.5 and −1.8 V in the same area as (**c**) (*V*_s_ = +1.2 V, *I*_t_ = 10 pA). (**e**) Averaged d*I*/d*V* spectra obtained from the regions indicated by blue (2L WS_2_), red (WS_2_/MoS_2_), and green (interface) squares in (**c**). (**f**) d*I*/d*V* color scale map calculated from the spatially resolved STS spectra along the white dotted line in (**c**).

To obtain additional insights into the origin of the conducting interface, STM/STS studies of the bilayer heterostructures were performed. Figure 4a shows a high-resolution STM image around the interface of the bilayer heterojunction. In this image, the left and right sides correspond to 2L WS_2_ and WS_2_/MoS_2_, respectively. This image shows a clear lattice composed of a surface WS_2_ layer and no structural defects around the interface. In Fig. 4b, FFT images of both the left and right sides of the STM image demonstrate six-fold symmetry spots with the same orientation. The wide-area STM image shows a dip of approximately 0.1 nm along the interface as indicated in black (Fig. 4c). This small dip can be mainly attributed to a noticeable difference in the electronic state at the interface. This is confirmed from the spatial change in the electronic structure in the same region, as visualized in the maps and spectra of d*I*/d*V* (where *I* and *V* are tunneling current and bias voltage, respectively), which is related to the local density of state (Fig. 4d–f). In particular, clear upshifts are observed around the interface in both the conduction band minimum (*E*_CBM_) and valence band maximum (*E*_VBM_) values. The bias voltage dependence d*I*/d*V* maps (Fig. 4d) indicate that only the interface shows high d*I*/d*V* values at bias voltages of −1.0 to −1.8 V, and therefore there is an upshift in *E*_VBM_ along the interface. The averaged d*I*/d*V* spectra show that the interface has a bandgap of approximately 1.5 eV (Fig. 4e), which is less than those of the 2L WS_2_ (2.1 eV) and WS_2_/MoS_2_ (2.0 eV). The mismatch between the lowest PL peak and the bandgap estimated by STM/STS is probably caused by several factors including the large exciton binding energies and the different measurement conditions such as temperature and substrates. Spatial changes in the band profile are observed within a 20 nm width zone around the interface as thresholds in the map using a d*I*/d*V* color scale (Fig. 4f). The *E*_VBM_ undergoes a larger upshift than the *E*_CBM_, thus narrowing the bandgap. As clearly shown in these maps and spectra, the present bilayer sample forms a type II semiconductor heterojunction with staggered bandgaps. The small bandgap of the WS_2_/MoS_2_ is also consistent with the present PL results (Supplementary Fig. S8) and with theoretical predictions based on first principles calculations^24^,^25^,^26^. These results indicate that the bandgap narrowing and/or the upshift in *E*_VBM_ lead to current increases around the interface during c-AFM measurements. In particular, the confining potential in the valence band could result in the accumulation of charge carriers along the interface and generate a 1D hole gas. In contrast, the potential barrier in the conduction band could allow control of the electron transport properties across the heterointerface by electric gating and thus would have applications to ultra-short channel switching devices.

## Discussion

**Figure 5:**
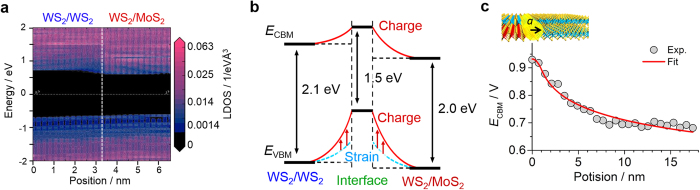
Proposed mechanism of band bending for the present bilayer-TMDC heterojunctions. (**a**) Calculated local density of state for the 2L WS_2_ and WS_2_/MoS_2_ heterojunction. (**b**) Schematic illustration of strain and piezoelectric polarization-induced band bending around the heterointerface. (**c**) Schematic illustration of the cylindrical space charge at the heterointerface and fitting results of spatial changes in *E*_CBM_ from the heterointerface in the 2L WS_2_ region.

Finally, we discuss the origin of the observed band profile around the heterointerface of bilayer samples. Possible origins could include an interface state due to Mo-S-W bonds, different electron affinities^24^, lattice strain, impurity doping, structural defects, changes in interlayer interaction^26^, and piezoelectric polarization^27^,^28^. From density functional calculations of the local density of state, we found that no mid-gap states are formed in the bandgaps of the 2L WS_2_ and MoS_2_/WS_2_ heterointerface (Fig. 5a). We also note that no large band offset has been observed for either the *E*_VBM_ or *E*_CBM_ in the STS analyses, which would be expected to be induced by a difference in electron affinity. We therefore propose that the band bending results from a combination of piezoelectric-charge-induced band upshift and strain-induced bandgap narrowing as a possible candidate, as shown in Fig. 5b, for the reasons detailed below.

In the case of the present bilayer interface, the *E*_VBM_ undergoes a remarkable upshift around the interface. This suggests that interlayer coupling could be one of the major factors affecting the bandgap narrowing because, for bilayer TMDCs, the *E*_VBM_ at the Γ point is predicted to be sensitive to the interlayer interactions^26^. The interlayer coupling can likely be changed by lattice strain, which generally has a maximum value at the heterointerface when the two materials have different lattice constants. In the present bilayer samples, the lattice constants and thermal expansion coefficients are still unknown but could have different values compared with these of monolayer samples. Importantly, the presence of interface strain can also explain the upshift in *E*_CBM_ in response to piezoelectric charges. Recently, the piezoelectric effect in TMDCs has been revealed by theoretical and experimental works^27^,^28^. Calculations predict that the 1L MoS_2_ and WS_2_ will have piezoelectric coefficients of 364 and 247 pC/m, respectively^27^. Using these values, the interface polarization, *P*_i_, can be readily estimated to be 117ε_11_ pC/m, where ε_11_ is the uniaxial strain. In the present work, the value of the interface space charge was evaluated from the measured band profile. Here, we assume that the *E*_CBM_ of the 2L WS_2_ region is up-shifted only by a fixed, negative cylindrical space charge with linear charge density λ, as pictured in Fig. 5c, with a spatially uniform dielectric environment. From familiar Gauss’s law, the potential distribution can be expressed as


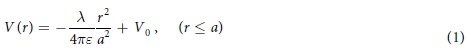



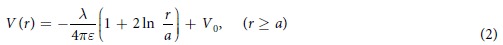


where *r* is the distance from the center, *a* is the radius of the cylindrical space charge, ε is an effective dielectric constant and *V*_0_ is the potential of the center. We found that this simple relation works well to reproduce the *E*_CBM_ profile (Fig. 5c) using the fitting parameters 1.0 nm for *a*, 4.4 pC/m for λ/ε and 0.93 V for *V*_0_. Note that this logarithmic dependence is a signature of 1D linear charges, which are different from conventional 2D plane charges. It is noteworthy that the resulting linear charge density is comparable to the value expected from piezoelectric polarization, (for example, *P*_i_ becomes 6 pC/m when ε_11_ is 0.05), if the interface has several percent lattice strain and if we use the calculated ε of 5.2 for bilayer MoS_2_^29^. Even though the accuracy of used values should be improved in the future, this supports the validity of our proposed model as a possible mechanism for the band bending.

It is also conceivable that the band bending is caused by other extrinsic factors, including the presence of impurities and structural defects such as sulfur vacancies, edges and grain boundaries. Recent STM/STS studies have reported the presence of mid-gap states at the heterointerface of 1L and 2L WSe_2_ (ref. 30), and the bandgap narrowing and the upshift of *E*_VBM_ and *E*_CBM_ for the grain boundaries of 1L MoS_2_ (ref. 31). A striking difference of the present work is that band bending was observed over a rather wide range of approximately 20 nm. In contrast, in previous studies, the local changes in *E*_VBM_ and *E*_CBM_ occur within an area of around 5 nm. This difference suggests that the present band bending is derived from another mechanism and is most likely affected by long-range lattice strain and/or fixed charges. Because the effects of such extrinsic factors are still unclear, further studies including the analysis of interface structures are necessary to reveal the origin of observed band bending.

In conclusion, we have observed the conductivity enhancement and band bending of a heterojunction interface composed of two-dimensional semiconductors. In particular, the bilayer heterojunction formed by 2L WS_2_ and WS_2_/MoS_2_ shows a unique band profile, as revealed by STM/STS. The present findings demonstrate that the atomic-layer heterojunctions provide an ideal system to realize tunable 1D electrical potential at heterointerfaces and should have applications in advanced electronics and opto-electronics. We note that 1D electron/hole gases and liquids exhibiting unique physical phenomena can be generated through carrier doping to the interface. Furthermore, it is suggested that these heterointerfaces allow us to control local confining potential and potential barrier via the piezoelectric effect, and so could be used as future switching devices, piezoelectric devices, and strain sensors. The interface fixed charges can also generate electrical field at the interface, which may be used to manipulate valley degree of freedom in TMDC-based optoelectronic devices^32^. The realization and application of such interface-related phenomena are exciting challenges in the field of atomic layer science.

## Methods

### Sample preparation

WS_2_ and MoS_2_ heterostructures were grown on Kish graphite (Type C, Covalent Materials Co.) or BN flakes using CVD, as reported previously^21^. The graphite crystals were mechanically exfoliated onto quartz substrates using Nitto tape (SPV-224). During the initial growth process, the substrate was placed in a quartz tube (3 cm diameter, 100 cm in length) together with WO_3_ (Aldrich, 99% purity, 15–50 mg) or MoO_2_ (Strem Chemicals, 99% purity, 5–15 mg) powder and sulfur flakes (Aldrich, 99.99% purity, 2 g). The quartz tube was then filled with Ar gas at a flow rate of 200 cm^3^/min. The temperature of the substrate and the WO_3_ or MoO_2_ powder was gradually increased to the sulfurization temperature (1100 °C) over 60 min using an electric furnace. When the substrate temperature was at the set point, the sulfur was heated at 160–180 °C for 15–30 min to supply sulfur vapor to the substrate, using a second electric furnace. Following the growth process, the quartz tube was immediately cooled using an electric fan. For the second growth, the sample was placed in a quartz boat, with the precursor (WO_3_ or MoO_2_) and sulfur flakes in a second quartz tube to avoid contamination. When the furnace temperature was at the set point, the sample was immediately introduced into the hot zone from the exterior of the furnace using a magnet in the quartz boat, and so exposed to the sulfur vapor. After the reaction had proceeded for 15–30 min, the sample was immediately cooled.

### Characterizations

Optical images were recorded with an optical microscope (Nikon, Eclipse-LV100D). Raman spectra were acquired using a micro-Raman spectroscope (Renishaw, inVia) with a 532 nm excitation laser. Topography and current images of the samples were obtained using atomic force microscopy (AFM, Shimadzu, SPM-9600). The current images were acquired at a bias voltage of −0.1 V. We note that the current is sensitive to the tip conditions because the conductivities between tip and samples are affected by various factors including tip shape and surface impurities of the tip. STM/STS assessments were carried out using a low-temperature STM system (UNISOKU, USM1200) operating at 83 K.

### Theoretical calculations

First principles calculations for projected local density of state were performed using the Atomistix Toolkit DFT code (Ver.2015.1) based on the nonequilibrium Green’s function method combined with density functional theory^33^. PBE-GGA was adopted as the exchange correlation functional and the basis set was double-zeta-polarized. A norm conserving pseudopotential was used for all atoms. The real-space grid cutoff was 75 Hartree. The geometry and the cell size were optimized until all forces were below 0.001 eV/Å and all stresses were less than 0.0001 eV/Å^3^. A vacuum gap of 15 Å was used in the direction normal to the material plane and there was no interaction between two materials separated by a vacuum. A schematic illustration of the simulation model is presented in Supplementary Fig. S10. The central region of the model included a heterojunction composed of a WS_2_ bilayer and vertically-stacked MoS_2_/WS_2_, with a central region consisting of a plane with dimensions 3.206 × 68.579 Å. An 8 × 100 *k*-point mesh was employed, and the central region was connected to two semi-infinite electrical leads on both sides.

## Additional Information

**How to cite this article**: Kobayashi, Y. *et al*. Modulation of electrical potential and conductivity in an atomic-layer semiconductor heterojunction. *Sci. Rep.* **6**, 31223; doi: 10.1038/srep31223 (2016).

## Supplementary Material

Supplementary Information

## References

[b1] Störmer H. L., Dingle R., Gossard A. C., Wiegmann W. & Sturge M. D. Two-dimensional electron gas at a semiconductor-semiconductor interface. Solid State Commun. 29, 705–709 (1979).

[b2] Takashi M., Satoshi H., Toshio F. & Kazuo N. A new field-effect transistor with selectively doped GaAs/n-Al_x_Ga_1-x_As heterojunctions. Jpn. J. Appl. Phys. 19, L225 (1980).

[b3] Dingle R., Störmer H. L., Gossard A. C. & Wiegmann W. Electron mobilities in modulation-doped semiconductor heterojunction superlattices. Appl. Phys. Lett. 33, 665–667 (1978).

[b4] Tsui D. C., Störmer H. L. & Gossard A. C. Two-dimensional magnetotransport in the extreme quantum limit. Phys. Rev. Lett. 48, 1559–1562 (1982).

[b5] Störmer H. L., Tsui D. C. & Gossard A. C. The fractional quantum Hall effect. Rev. Mod. Phys. 71, S298–S305 (1999).

[b6] Ohtomo A. & Hwang H. Y. A high-mobility electron gas at the LaAlO_3_/SrTiO_3_ heterointerface. Nature 427, 423–426 (2004). 14749825 10.1038/nature02308

[b7] Reyren N. . Superconducting interfaces between insulating oxides. Science 317, 1196–1199 (2007). 17673621 10.1126/science.1146006

[b8] Levendorf M. P. . Graphene and boron nitride lateral heterostructures for atomically thin circuitry. Nature 488, 627–632 (2012). 22932386 10.1038/nature11408

[b9] Sutter P., Cortes R., Lahiri J. & Sutter E. Interface formation in monolayer graphene-boron nitride heterostructures. Nano Lett. 12, 4869–4874 (2012). 22871166 10.1021/nl302398m

[b10] Miyata Y. . Fabrication and characterization of graphene/hexagonal boron nitride hybrid sheets. Appl. Phys. Express 5, 085102 (2012).

[b11] Liu Z. . In-plane heterostructures of graphene and hexagonal boron nitride with controlled domain sizes. Nature Nanotech. 8, 119–124 (2013). 10.1038/nnano.2012.256 23353677

[b12] Liu L. . Heteroepitaxial growth of two-dimensional hexagonal boron nitride templated by graphene edges. Science 343, 163–167 (2014). 24408431 10.1126/science.1246137

[b13] Gong Y. . Vertical and in-plane heterostructures from WS_2_/MoS_2_ monolayers. Nature Mater. 13, 1135–1142 (2014). 25262094 10.1038/nmat4091

[b14] Duan X. . Lateral epitaxial growth of two-dimensional layered semiconductor heterojunctions. Nature Nanotech. 9, 1024–1030 (2014). 10.1038/nnano.2014.222 PMC12049235 25262331

[b15] Huang C. . Lateral heterojunctions within monolayer MoSe_2_/WSe_2_ semiconductors. Nature Mater. 13, 1096–1101 (2014). 25150560 10.1038/nmat4064

[b16] Kobayashi Y., Mori S., Maniwa Y. & Miyata Y. Bandgap-tunable lateral and vertical heterostructures based on monolayer Mo_1−x_W_x_S_2_ alloys. Nano Res. 8, 3261–3271 (2015).

[b17] Yoshida S. . Microscopic basis for the band engineering of Mo_1-x_W_x_S_2_-based heterojunction. Sci. Rep. 5, 14808 (2015). 26443124 10.1038/srep14808 PMC4595798

[b18] Li M.-Y. . Epitaxial growth of a monolayer WSe_2_-MoS_2_ lateral p-n junction with an atomically sharp interface. Science 349, 524–528 (2015). 26228146 10.1126/science.aab4097

[b19] Chhowalla M. . The chemistry of two-dimensional layered transition metal dichalcogenide nanosheets. Nature Chem. 5, 263–275 (2013). 23511414 10.1038/nchem.1589

[b20] Wang Q. H., Kalantar-Zadeh K., Kis A., Coleman J. N. & Strano M. S. Electronics and optoelectronics of two-dimensional transition metal dichalcogenides. Nature Nanotech. 7, 699–712 (2012). 10.1038/nnano.2012.193 23132225

[b21] Kobayashi Y. . Growth and optical properties of high-quality monolayer WS_2_ on graphite. ACS Nano, 4056–4063 (2015). 25809222 10.1021/acsnano.5b00103

[b22] Zhao W. . Origin of indirect optical transitions in few-layer MoS_2_, WS_2_, and WSe_2_. Nano Lett. 13, 5627–5634 (2013). 24168432 10.1021/nl403270k

[b23] Zhao W. . Evolution of electronic structure in atomically thin sheets of WS_2_ and WSe_2_. ACS Nano 7, 791–797 (2013). 23256505 10.1021/nn305275h

[b24] Kośmider K. & Fernández-Rossier J. Electronic properties of the MoS_2_-WS_2_ heterojunction. Phys. Rev. B 87, 075451 (2013).

[b25] Terrones H., López-Urías F. & Terrones M. Novel hetero-layered materials with tunable direct band gaps by sandwiching different metal disulfides and diselenides. Sci. Rep. 3, 1549 (2013). 23528957 10.1038/srep01549 PMC3607896

[b26] Komsa H.-P. & Krasheninnikov A. V. Electronic structures and optical properties of realistic transition metal dichalcogenide heterostructures from first principles. Phys. Rev. B 88, 085318 (2013).

[b27] Duerloo K.-A. N., Ong M. T. & Reed E. J. Intrinsic piezoelectricity in two-dimensional materials. J. Phys. Chem. Lett. 3, 2871–2876 (2012).

[b28] Wu W. . Piezoelectricity of single-atomic-layer MoS_2_ for energy conversion and piezotronics. Nature 514, 470–474 (2014). 25317560 10.1038/nature13792

[b29] Cheiwchanchamnangij T. & Lambrecht W. R. L. Quasiparticle band structure calculation of monolayer, bilayer, and bulk MoS_2_. Phys. Rev. B 85, 205302 (2012).

[b30] Zhang C. . Visualizing band offsets and edge states in bilayer-monolayer transition metal dichalcogenides lateral heterojunction. Nature Commun. 7, 10349 (2016). 26778119 10.1038/ncomms10349 PMC4735610

[b31] Huang Y. L. . Bandgap tunability at single-layer molybdenum disulphide grain boundaries. Nature Commun. 6, 6298 (2015). 25687991 10.1038/ncomms7298

[b32] Zhang Y. J., Oka T., Suzuki R., Ye J. T. & Iwasa Y. Electrically switchable chiral light-emitting transistor. Science 344, 725–728 (2014). 24790028 10.1126/science.1251329

[b33] Stokbro K., Blom A. & Smidstrup S. Atomistic simulation of a III-V p-i-n junction: Comparison of density functional and tight-binding approaches. International Conference on Simulation of Semiconductor Processes and Devices (SISPAD). 380–383 (2013).

