# Supplementary material for: Modulation of electrical potential and conductivity in an atomic-layer semiconductor heterojunction
Source: Sci Rep. 2016 Aug 12;6:31223. doi: 10.1038/srep31223 (PMC4981840; doi:10.1038/srep31223)
Supplement: Supplementary Information [file srep31223-s1.pdf]

Supporting Information

Modulation of electrical potential and conductivity in an atomic-layer semiconductor heterojunction

Yu Kobayashi[1], Shoji Yoshida[2], Ryuji Sakurada[2], Kengo Takashima[3], Takahiro Yamamoto[3,4], Tetsuki Saito[1], Satoru Konabe[5], Takashi Taniguchi[6], Kenji Watanabe[6], Yutaka Maniwa[1], Osamu Takeuchi[2], Hidemi Shigekawa[2], Yasumitsu Miyata[1,7,]*

[1]Department of Physics, Tokyo Metropolitan University, Hachioji, Tokyo 192-0397, Japan

[2]Faculty of Pure and Applied Sciences, University of Tsukuba, Tsukuba, Ibaraki 305-8573, Japan

[3]Department of Electrical Engineering, Graduate School of Engineering, Tokyo University of Science, Katsushika, Tokyo 125-8585, Japan

[4]Department of Liberal Arts, Faculty of Engineering, Tokyo Univrsity of Science, Katsushika, Tokyo 125-8585, Japan

[5]Research Institute for Science and Technology, Tokyo University of Science, Katsushika, Tokyo 125-8585, Japan

[6]National Institute for Materials Science, Tsukuba, Ibaraki 305-0044, Japan

[7]JST-PRESTO, Kawaguchi, Saitama 332-0012, Japan

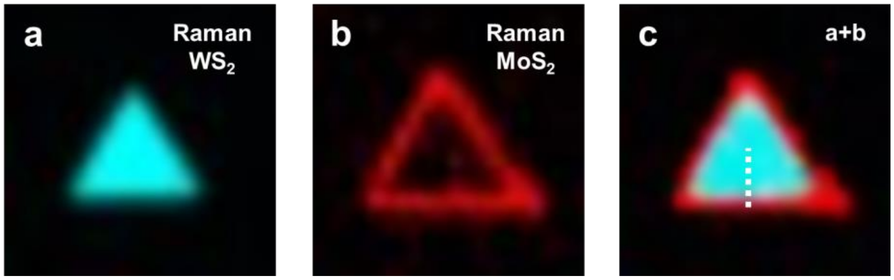

Figure S1. Raman intensity maps of (a) 2LA and E' modes of WS_2 (cyan), (b) E' mode of MoS_2 (red), and (c) a combination of (a) and (b).

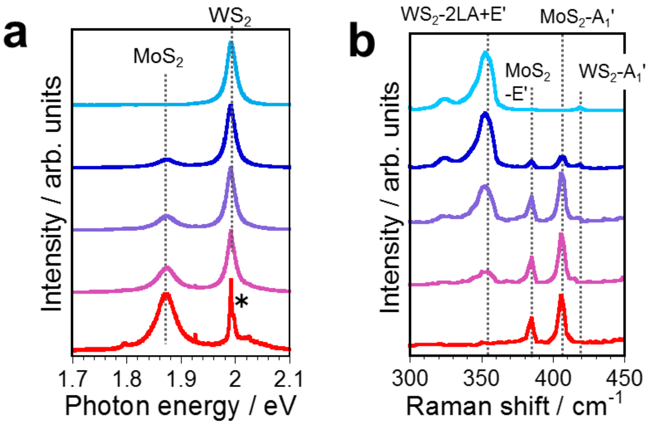

Figure S2. (a) PL and (b) Raman spectra acquired at five points from top to bottom along the dotted lines in Figs. 2d and S1c, respectively. The asterisk indicates the 2D Raman band of the graphite substrate.

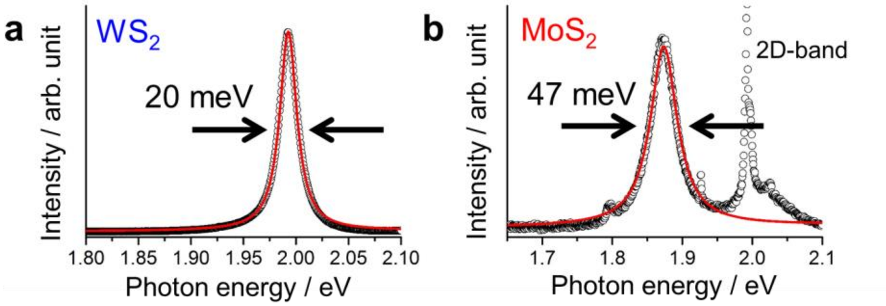

Figure S3. PL spectra of monolayer (a) WS_2 and (b) MoS_2 grown on graphite. The peak at approximately 2.0 eV corresponds to the 2D Raman band of the graphite substrate.

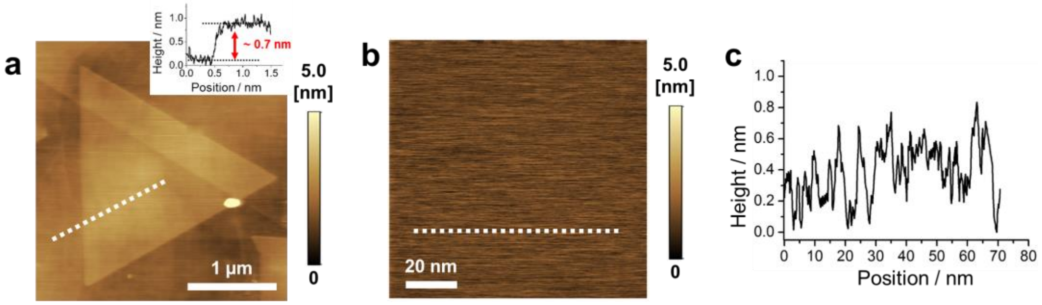

Figure S4. Topographic images of (a) the grain and (b) the expanded area. The inset shows the height profile along the dotted line in (a). (c) Height profile along the dotted line in (b).

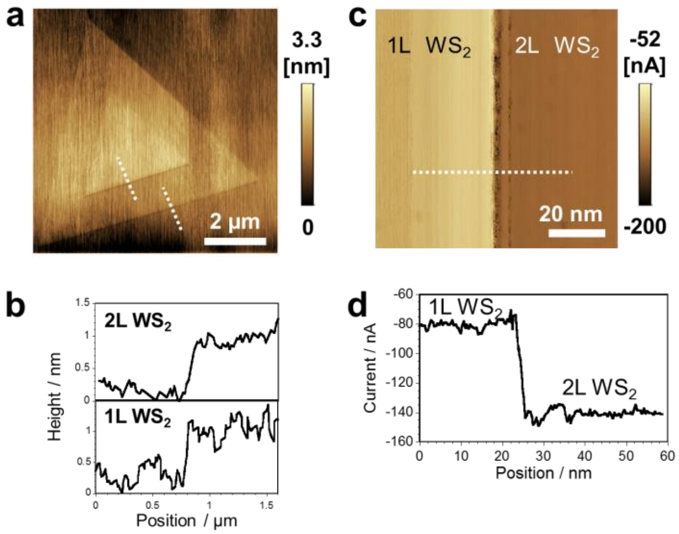

Figure S5. (a) Topographic image, (b) height profile, (c) current image, and (d) current profile of a WS_2 grain with a 1L-2L WS2 heterojunction. Height and current profiles were obtained along the dotted lines in (a) and (c), respectively.

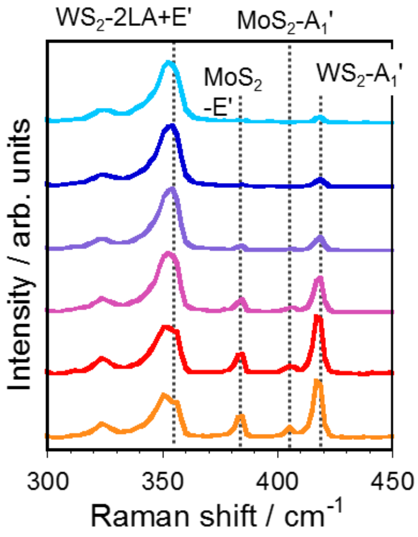

Figure S6. Raman spectra acquired at six points from top to bottom along the dotted line in Fig. 3e.

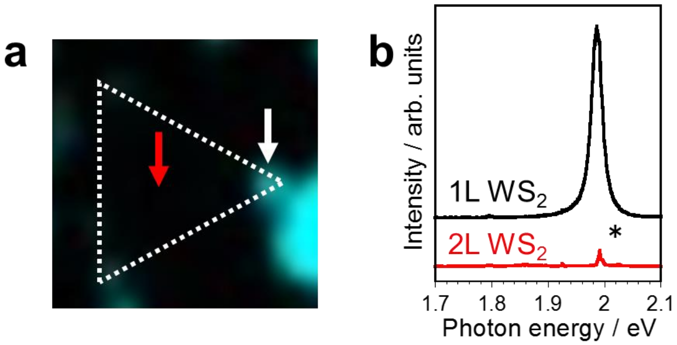

Figure S7. (a) PL intensity map of monolayer WS_2 around the bilayer heterostructure shown in Fig. 3. (b) PL spectra acquired at the bilayer (red) and monolayer (white) regions indicated by arrows. The peak indicated by an asterisk corresponds to the 2D Raman band of the graphite substrate.

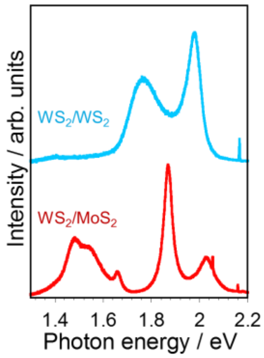

Figure S8. PL spectra of 2L WS_2 (blue) and WS_2/MoS_2 (red) vertical heterostructures grown on BN flakes.

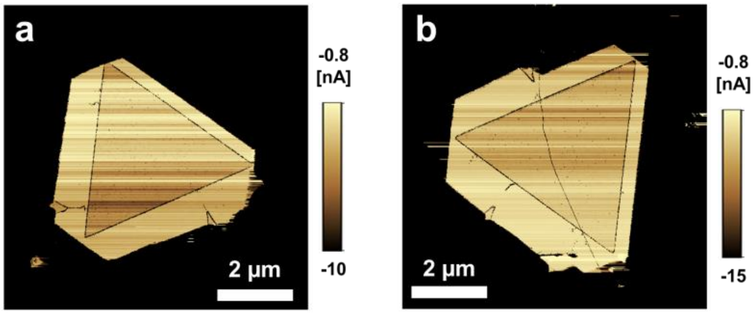

Figure S9. (a and b) Current images of two different grains with bilayer heterostructures.

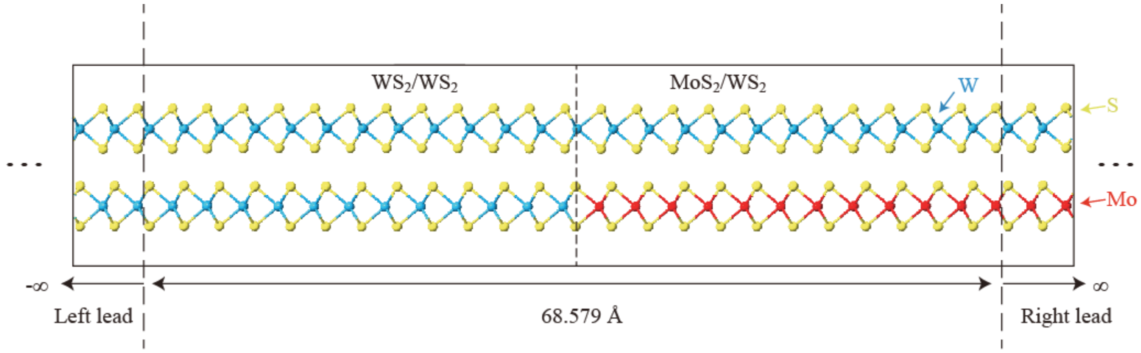

Figure S10. Numerical calculation model for a bilayer TMDC heterojunction.
